# Association Between Volatile Organic Compound Metabolites in Urine and Adult Lung Function: Evidence From NHANES 2011–2012

**DOI:** 10.1111/crj.70192

**Published:** 2026-05-01

**Authors:** Qian Liu, Yanpeng Fu, Yuchen Tao, Wenyu Chen, Yue Wang, Haiyan Yin, Qianqian Yang

**Affiliations:** ^1^ Department of Otorhinolaryngology Head and Neck Surgery First Affiliated Hospital of Soochow University Suzhou Jiangsu China; ^2^ Department of Otorhinolaryngology Head and Neck Surgery Second Affiliated Hospital of Nanchang University Nanchang Jiangxi China; ^3^ Department of Pathology First Affiliated Hospital of Soochow University Suzhou Jiangsu China; ^4^ Interventional Cardiology Department Second Affiliated Hospital of Nanchang University Nanchang Jiangxi China; ^5^ Department of Otorhinolaryngology—Head Neck Surgery Ningbo First Hospital Ningbo Zhejiang China; ^6^ Jining Key Laboratory of Pharmacology, School of Basic Medical Science Jining Medical University Jining Shandong China

**Keywords:** Bayesian kernel machine regression (BKMR) model, fractional exhaled nitric oxide (FeNO), lung function, tobacco smoke, volatile organic compounds (VOCs)

## Abstract

**Objectives:**

Volatile organic compounds (VOCs) have been reported to be associated with adverse respiratory outcomes during daily exposure at low levels. However, the main contributors among the numerous VOCs and tobacco as the possible sources of VOCs in the general population have not been fully revealed. Here, we aimed to examine the association between urinary VOCs metabolites (UM‐VOCs) mixtures and adult lung health.

**Methods:**

We used data from the National Health and Nutrition Examination Survey (NHANES) for the years 2011–2012 and aimed at 16 UM‐VOCs. Initially, we used linear regression to evaluate the association between a single compound and lung health. Next, we utilized the BKMR model to assess the overall effect and attempt to understand the potential modifying effects of smoking.

**Results:**

Our linear regression analysis suggested that the majority of the UM‐VOCs were inversely related to lung function. In multivariable linear regression models, each one‐unit increase in natural log‐transformed urinary concentrations (μg/mmol Cr) of AMCC, CYMA, MHBMA3, 3HPMA, AAMA, and MA was associated with an absolute decrease in 1 s/forced vital capacity (FEV1/FVC) ratio of 3.5, 1.6, 2.3, 3.0, 3.2, and 4.6 percentage points, respectively (all *p* < 0.05). Moreover, 3HPMA and HPMMA were positively correlated with wheeze symptom, while 2MHA and 3MHA + 4MHA were negatively correlated with fractional exhaled nitric oxide (FeNO), respectively. BKMR model results showed that UM‐VOC mixtures exhibited a negative correlation with FEV1/FVC and FeNO. Urinary AMCC and CYMA were identified as the most important contributors to the decline in FEV1/FVC and FeNO, respectively. Subsequently, we found UM‐VOCs of higher concentration in smokers, and stratified BKMR analyses revealed an effect modification by smoking status.

**Conclusions:**

Using BKMR, we found that higher concentrations of urinary metabolites of VOCs (UM‐VOCs) were associated with lower FEV_1_/FVC ratios and lower FeNO levels in a population‐based study. This association was primarily observed among smokers, suggesting that tobacco smoke may be a significant contributor to VOC exposure.

## Graphical abstract

Using BKMR, we observed the joint effects of 16 UM‐VOCs on adult lung function decline. Urinary AMCC and CYMA were identified as the most important contributors to the decline in FEV1/FVC and FeNO, respectively. Tobacco smoke may be the main source of VOCs exposure.

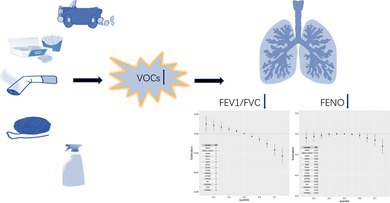

## 1 Introduction

VOCs are ubiquitously existed in the environment, characterized by low boiling points and high vapor pressure at room temperatures. Tobacco smoke, vehicle exhaust, daily‐consumed foodstuffs, textiles, and household cleaning products are probably the common sources of human exposure to VOCs [1, 2]; however, direct evidence, especially based on large population samples, was lacking. For the general population, inhalation is the main route of exposure to VOCs [3, 4]. VOCs, due to their low polarity and poor water solubility, can accumulate in biological tissues over time following repeated exposure [5] and may adversely affect the respiratory system, contributing to asthma, chronic obstructive pulmonary disease, and other lung diseases [6, 7, 8, 9]. Recent epidemiological evidence further supports these associations, including studies linking VOC exposure to impaired lung function and increased respiratory morbidity and mortality [10]. FeNO, originating from the airway epithelium, is an important marker of airway inflammation [11, 12], especially for type 2, which is useful for asthma diagnosis [13]. But we also heard the opposite sound produced recently [14, 15], which makes the precise relationship between VOCs and lung function or FeNO confusing. Furthermore, our study specifically examined the effects of tobacco smoking on the lungs, as the lungs are the primary target organ affected, making up tobacco‐related complications [16]. Additionally, the relationship between smoking and VOCs was not fully understood.

Most previous studies measure exposure to VOCs primarily using environmental samples, blood, or breath. However, assessment of exposure to VOCs by sampling air is not equivalent for measuring internal absorbed dose [17, 18] and breath biochemistry also shows a high variability [19, 20]. Blood is the most accurate indicator of physiological state, but the blood draw is an invasive examination with low adherence. Compared with blood, there is a longer biological half‐life of VOC metabolites in urine, and they are more stable without volume and frequency limitation [21]. But fewer studies have examined the relationship between urinary VOC metabolites and lung function, particularly in large population‐based settings. Recent studies integrating air pollution exposure, lung function, and airway inflammation markers have begun to provide more comprehensive insights into these relationships [22]. And most current studies only focus on single component, and the potential interaction in mixture is frequently neglected.

This study, therefore, aimed to investigate the associations of urinary VOC metabolites with lung function and FeNO among US adults using data from the National Health and Nutrition Examination Survey (NHANES) 2011–2012. Our study offers several novel contributions to the existing literature: First, unlike most previous studies that focused on individual VOCs, we employed Bayesian Kernel Machine Regression (BKMR) to evaluate the joint effects of 16 UM‐VOCs as a mixture and identify the most influential components. Second, we simultaneously assessed both lung function parameters (FEV1, FVC, and FEV1/FVC) and an airway inflammation marker (FeNO) to provide a more comprehensive evaluation of respiratory health. Third, we conducted stratified analyses by smoking status to explore whether the associations between VOC exposure and respiratory outcomes differ between smokers and nonsmokers, which may provide insight into the potential role of tobacco smoke as a source of VOC exposure. We hypothesized that environmental VOC exposure is associated with reduced lung function and tobacco smoke may contribute to VOC exposure.

## 2 Methods

### 2.1 Study Population

We used the dataset (*N* = 9756) from 2011–2012 NHANES, a series of population‐based surveys designed to investigate the health and nutrition of the U.S. population, and excluded participants with uncompleted information of urinary VOCs metabolites (UM‐VOCs), lung function testing or FeNO. Additionally, we removed the participants whose covariate data were missing, including education, poverty index ratio, and alcohol‐drinking history among others. Finally, 1020 participants, ages 20 to 79 years, who are adults more apt to tobacco exposure, were included in the analyses for the associations of UM‐VOCs with lung function and FeNO (Figure S1). All procedures were approved by the NCHS Ethic Review Board, and all participants signed informed consent.

### 2.2 Urinary VOC Metabolites Measurement

These metabolites were quantified as previously indicated [21]. In our study, 16 VOC metabolites with detectable frequencies ≥ 75% were selected for inclusion. Table S1 shows the parent compounds and metabolites included in our analysis, and the detectable frequencies for all 16 UM‐VOCs. For the 16 UM‐VOCs assessed in this study, values below the limit of detection (LOD) were replaced with LOD/√2. Additionally, the corresponding daily urine creatinine concentration for each individual was used to standardize the daily urinary concentration of each VOC metabolite for each individual. Results are given in units of μg/mmol Cr.

### 2.3 Lung Function and FeNO Measurement

Procedures are described in detail on the website of NHANES (https://www.cdc.gov/nchs/nhanes/index.htm). The lung function test findings are presented as absolute values for FEV1, representing the movement of air through the larger airway, and FVC, revealing static lung volume, in milliliters, as well as the FEV1 to FVC ratio, reflecting the caliber of larger airways [23, 24].

### 2.4 Asthma Assessment

Asthma was defined by positive answers to the question: “Has a doctor or other health professional ever told you that you have asthma?” and a positive answer to the question “do you still have asthma” are further defined as current asthma. Wheeze was defined by a positive answer to the question: “In the past 12 months, have you had wheeze or whistling in your chest?”

### 2.5 Demographic Covariates

In order to gather demographic data on gender, age, race/ethnicity, drinking status, poverty income ratio (PIR), body mass index (BMI), and education, the participants completed standardized questionnaires from professional interviewers in their residences. Race/ethnicity was categorized as non‐Hispanic White (NHW), non‐Hispanic Black (NHB), and others. Drinking status was defined by positive answers to the question: “Had at least 12 alcohol drinks/1 year?” PIR was calculated by dividing the household income by the poverty guidelines for the given survey year. Height and weight were measured by a trained health technician following a standardized protocol, and BMI was calculated. Serum cotinine levels were measured and divided into < 10 and ≥ 10 ng/mL, reflecting the status of smoking (nonsmokers defined as those with serum cotinine values of < 10 ng/mL, smokers defined as those with serum cotinine values of ≥ 10 ng/mL). Normally, values are expressed as mean ± SD (continuous variables) or percentage (categorical variables).

### 2.6 Statistics

The associations of concentrations of each UM‐VOC as continuous variables with lung function, FeNO and asthma in adult were firstly analyzed by conventional multivariable linear regression. Logistic and linear models were used to estimate dichotomous and continuous outcomes, respectively. Regression models were built for natural log‐transformed values of each UM‐VOC listed above due to the right‐skewed distribution of the data. For the interpretation of percentage changes in FEV1/FVC, because UM‐VOC concentrations were natural log‐transformed, the regression coefficient (*β*) represents the absolute change in the outcome variable (FEV1/FVC, expressed as a percentage) associated with a one‐unit increase in ln‐UM‐VOC concentration (μg/mmol Cr). For example, a *β* of −0.035 indicates that a one‐unit increase in ln‐UM‐VOC concentration is associated with a 3.5 percentage point decrease in FEV1/FVC. Spearman correlation analysis was then utilized to estimate the associations between pairwise chemical concentrations. To correct for multiple comparisons, we used the modified effective number proposed by Li et al. [25], as detailed below. We extracted the effective number of tests (*Meff*) by Equation (1), where $\lambda_{i}$ represents the eigenvalues of the similarity matrix. The *Meff* was 10 in this study. Then we performed a *Meff*‐based false discovery rate (FDR) procedure to adjust *p* values by Equation (2).

(1) $$\left\{\begin{array}{c} M_{\mathrm{eff}}=\sum_{i=1}^{M}f\left(|\lambda_{i}|\right)\\ f\left(x\right)=I\left(x\geq 1)\right.+\left(x-\left\lfloor \left.x\right\rfloor \right.\right),\;x\geq 0 \end{array}\right..$$

(2) $$\left\{\begin{array}{c} k=\max_{1\leq i\leq M}\left\{i:p_{\left(i\right)}\leq \frac{q}{\text{Meff}}+\frac{i-1}{M+1}\left(q-\frac{q}{\text{Meff}}\right)\right\}. \end{array}\right.$$

*p* value < 0.05 was regarded as a robust result. And the data were shown as *β* coefficients and 95% confidence intervals (CI). The covariates included in all models were the following: age, race/ethnicity, gender, smoking status, drinking status, BMI, PIR, education, and urine creatinine. These covariates were selected because we considered them to be clinically relevant confounders of the association between UM‐VOCs and respiratory outcomes based on the results of previous studies [26, 27, 28].

To our knowledge, the BKMR model offers greater flexibility for evaluating the combined impact of multiple pollutants and identifying the potential interactions and nonlinear effects of mixture exposures, as well as in interpreting exposure–response relationship for each ingredient in the mixture [29]. Thus, we performed a secondary analysis using BKMR to compare with our primary analysis.

Specifically, to investigate the overall effects, we employed BKMR to assess the changes in the outcome variables when all the compounds were fixed at a particular percentile compared with those fixed at their median values. To determine the proportionate contribution of each mixture component to the overall mixture impact, we computed the conditional posterior inclusion probability (PIP) [29]. PIPs were 0 to 1, and greater PIPs indicate a more significant variable. Then, with all other chemicals set to their median values, we investigated the univariate correlations between individual chemicals and lung health. Considering the potential modifying effects of smoking and asthma, a stratification analysis was also performed in the mixture of UM‐VOCs. And we compared relative UM‐VOCs concentrations among smokers and nonsmokers by Wilcoxon test. It should be noted that smokers and individuals with asthma may have different baseline lung function and airway inflammation levels regardless of UM‐VOC exposure. In our stratified BKMR analyses, we did not additionally adjust for baseline lung function, as these factors may be intermediate variables or consequences of smoking/asthma status rather than confounders. Therefore, the stratified analyses should be interpreted as descriptive of exposure–response patterns within subgroups rather than formal tests of effect modification. Finally, we used bivariate dose–response curves to examine potential interactions among 16 chemicals in mixtures by visualizing the exposure–response function of a single chemical when another chemical was fixed at varying (25th, 50th, and 75th) percentiles, and all the other chemicals were fixed at the median. To test the stability of the study results, sensitivity analysis methods were performed with or without adjusted creatinine levels by BKMR. All statistical computations were done using R studio (R version 3.4.1). Statistical significance was defined as *p* < 0.05 (two tails). It is important to note that conventional multivariable linear regression and the BKMR model address different research questions. Linear regression estimates the independent association of each individual UM‐VOC with health outcomes, adjusting for other compounds. In contrast, BKMR evaluates the joint effect of the entire mixture while accounting for correlations and potential nonlinear relationships among components and identifies the relative contribution of each chemical within the mixture. These two analytical approaches are complementary, providing a more comprehensive understanding of the exposure–response relationships. Therefore, this study aims to evaluate VOC exposure as a mixture using BKMR, while simultaneously examining lung function and airway inflammation (FeNO) in a population‐based setting.

## 3 Results

### 3.1 Study Population

Table 1 tabulated the socio–demographic characteristics of the study population. There were 1020 participants in all who were eligible and had exposure and outcome information available (Figure S1). Population‐weighted mean age was 44.86 years and the mean BMI was 28.69 kg/m^2^. 49.5% of the population were female, and non‐Hispanic White made up the majority of the population. Roughly 14.3% of participants were below the poverty line. More than half of the population was highly educated or had no alcohol history.

**TABLE 1:** Participant characteristics (*N* = 1020) in NHANES 2011–2012.

Characteristic	Participant
Age, mean (SD)	44.86 (15.68)
Gender, *n* (%)	
Men	546 (50.5)
Women	474 (49.5)
Race, *n* (%)	
Non‐Hispanic Black	266 (10.5)
Non‐Hispanic White	393 (69.4)
Other	361 (20.1)
Education, *n* (%)	
Less than high school	180 (12.2)
High school graduate or GED	207 (20.0)
Some college or AA	318 (30.7)
College graduate or more	315 (37.1)
PIR, *n* (%)	
≤ 1	217 (14.3)
1–3	397 (35.6)
≥ 3	406 (50.1)
History of drinking, *n* (%)	
No	795 (83.8)
Yes	225 (16.2)
Wheeze, *n* (%)	
No	901 (88.3)
Yes	119 (11.7)
Asthma, *n* (%)	
No	871 (84.8)
Yes	149 (15.2)
Current asthma, *n* (%)	
No	946 (91.4)
Yes	74 (8.6)
BMI, mean (SD)	28.69 (6.73)
Urinary creatinine, mean (SD)	115.89 (80.84)
Serum cotinine, mean (SD)	49.75 (121.03)
FeNO, mean (SD)	16.20 (13.98)
FVC, mean (SD)	4151.40 (1114.02)
FEV1, mean (SD)	3236.81 (917.11)
FEV1/FVC (%), mean (SD)	0.78 (0.08)

*Note:* Mean, SD, and percentages are weighted.

Abbreviations: BMI, body mass index; FeNO, fractional exhaled nitric oxide; FEV1, forced expiratory volume in the first second of forced vital capacity; FVC, forced vital capacity; PIR, poverty–income ratio.

### 3.2 Effects of the Levels of UM‐VOCs on Lung Health

We used the conventional multivariable linear regression in the primary analyses. Tables 2 and S2 illustrate the relationship between each UM‐VOC concentration and lung function, FeNO, and asthma, respectively. Certain *p* values remained statistically significant after correcting as shown in Table 2. Statistically significant inverse associations were observed between several UM‐VOCs and lung function or FeNO. Every natural log‐fold increase in the concentrations of urinary AMCC, CYMA, MHBMA3, 3HPMA, AAMA, and MA corresponded to a 3.5%, 1.6%, 2.3%, 3%, 3.2%, and 4.6% (*p* < 0.05) decrease in FEV1/FVC, respectively. Moreover, 2MHA and 3MHA + 4MHA were negatively correlated with FeNO, whereas 3HPMA and HPMMA were positively correlated with wheeze symptoms. But no obvious association was found between individual UM‐VOC and asthma (Table S2).

**TABLE 2:** Association of the concentrations of urinary VOCs metabolites with FEV1, FVC, FEV1/FVC, FeNO, and wheeze (linear regression models).

Metabolites	FEV1		FVC		FEV1/FVC		FeNO		Wheeze	
	*β* (95% CI)	Adjusted *p*	*β* (95% CI)	Adjusted *p*	*β* (95% CI)	Adjusted *p*	*β* (95% CI)	Adjusted *p*	*β* (95% CI)	Adjusted *p*
2MHA	−39 (−72, −6.2)	0.138	−14 (−75, 48)	0.840	−0.011 (−0.024, 0.001)	0.242	−0.098 (−0.13, −0.062)	0.039*	1.3 (1.1, 1.6)	0.108
3MHA + 4MHA	−51 (−90, −12)	0.118	−27 (−91, 36)	0.858	−0.012 (−0.026, 0.002)	0.226	−0.13 (−0.17, −0.093)	0.025*	1.2 (0.98, 1.5)	0.236
AAMA	−83 (−130, −35)	0.095	−22 (−79, 36)	0.865	−0.032 (−0.046, −0.019)	0.040*	−0.057 (−0.14, 0.023)	0.336	1.7 (1.2, 2.4)	0.097
AMCC	−22 (−90, 46)	0.592	58 (−18, 130)	0.750	−0.035 (−0.048, −0.022)	0.039*	−0.0094 (−0.11, 0.091)	0.920	1.5 (1.1, 2)	0.125
ATCA	−90 (−150, −35)	0.094	−89 (−160, −16)	0.741	−0.0092 (−0.02, 0.002)	0.222	−0.039 (−0.097, 0.019)	0.336	1.2 (0.9, 1.7)	0.345
BMA	−28 (−83, 28)	0.440	−12 (−78, 53)	0.775	−0.0093 (−0.021, 0.002)	0.227	0.056 (0.0025, 0.11)	0.274	1.1 (0.87, 1.3)	0.579
BPMA	−23 (−49, 4.1)	0.226	−17 (−41, 6.7)	0.680	−0.005 (−0.013, 0.003)	0.325	0.033 (−0.006, 0.073)	0.336	1.1 (0.89, 1.3)	0.467
CEMA	−82 (−180, 16)	0.217	−65 (−160, 29)	0.616	−0.017 (−0.042, 0.008)	0.291	−0.043 (−0.14, 0.057)	0.508	1.6 (0.94, 2.7)	0.228
CYMA	−30 (−52, −8.3)	0.117	4 (−29, 37)	0.826	−0.016 (−0.022, −0.011)	0.036*	−0.071 (−0.11, −0.031)	0.110	1.3 (1.2, 1.5)	0.055
DHBMA	−160 (−240, −81)	0.106	−120 (−220, −20)	0.490	−0.032 (−0.052, −0.012)	0.076	−0.0032 (−0.13, 0.12)	0.963	1.8 (1.2, 2.9)	0.127
2HPMA	−43 (−84, −3.1)	0.160	−18 (−70, 35)	0.853	−0.018 (−0.031, −0.005)	0.113	−0.057 (−0.1, −0.013)	0.187	1.1 (0.92, 1.4)	0.338
3HPMA	−130 (−190, −70)	0.138	−96 (−180, −9.9)	0.428	−0.03 (−0.044, −0.017)	0.042*	−0.057 (−0.15, 0.033)	0.345	1.7 (1.4, 2.1)	0.047*
MA	−81 (−140, −26)	0.110	19 (−69, 110)	0.791	−0.046 (−0.069, −0.024)	0.040*	−0.075 (−0.17, 0.022)	0.321	1.5 (1, 2.3)	0.208
MHBMA3	−70 (−110, −35)	0.081	−30 (−75, 15)	0.579	−0.023 (−0.034, −0.013)	0.036*	−0.11 (−0.17, −0.043)	0.111	1.5 (1.2, 1.9)	0.056
PGA	−11 (−100, 80)	0.827	33 (−76, 140)	0.837	−0.015 (−0.043, 0.013)	0.357	−0.084 (−0.19, 0.021)	0.330	1.3 (0.85, 1.9)	0.361
HPMMA	−58 (−110, −3.8)	0.149	−18 (−97, 61)	0.897	−0.022 (−0.041, −0.002)	0.172	−0.068 (−0.14, 0.008)	0.334	1.7 (1.4, 2)	0.046*

Abbreviations: FeNO, fractional exhaled nitric oxide; FEV1, forced expiratory volume in the first second of forced vital capacity; FVC, forced vital capacity.

* *p* < 0.05.

### 3.3 Joint Effects of 16 UM‐VOCs on Lung Health

**FIGURE 1:**
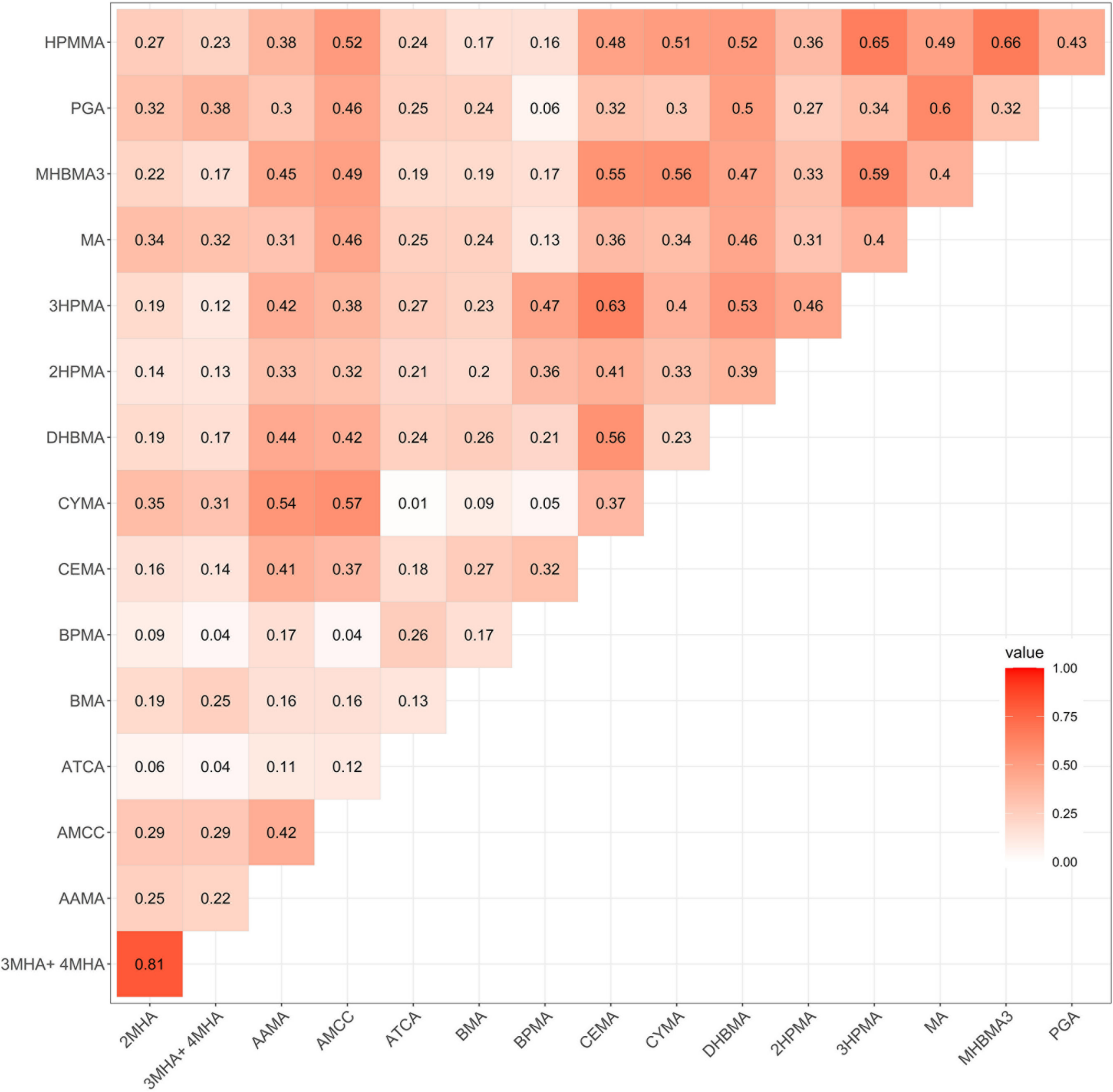
Spearman coefficients among 16 UM‐VOCs concentrations.

Figure 1 shows the spearman coefficients among 16 UM‐VOCs concentrations. We observed moderate to high correlations within the most of UM‐VOCs whereas only ATCA, BMA, and BPMA show a “very weak” correlation with the remaining UM‐VOCs according to the heat map of Spearman correlations. Due to the apparent correlation between the compounds, we used the BKMR method to compare our primary analysis results.

**FIGURE 2:**
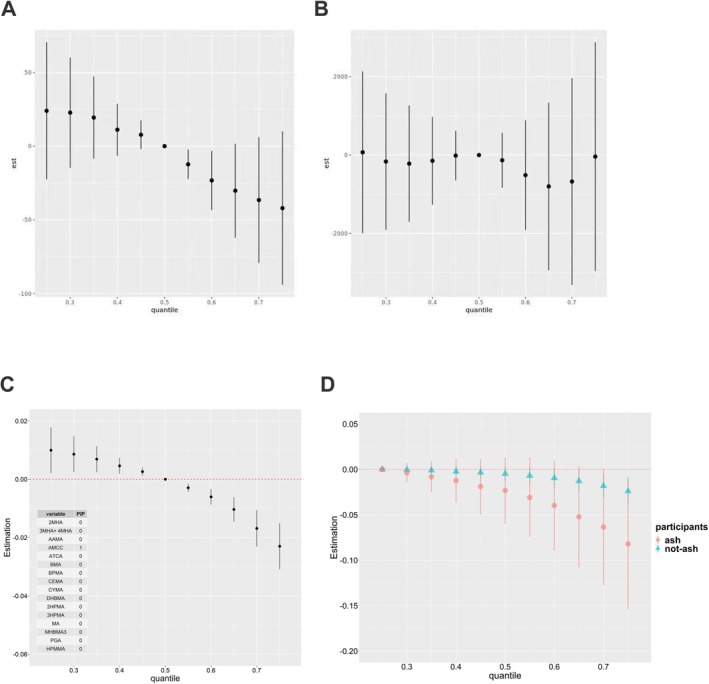
Overall effect of the 16‐UM‐VOC mixture on lung function. The UM‐VOCs mixture was associated with a significant decrease in FEV1/FVC when all chemicals were at or above the 60th percentile compared with their median values (C), whereas no significant overall associations were observed for FEV1 (A) or FVC (B). Posterior inclusion probabilities (PIPs) for each component in the FEV1/FVC model are shown, with AMCC identified as the dominant contributor (PIP = 1.0) (C). Stratified analyses by asthma for FEV1/FVC by BKMR model. Red indicates asthma, and green represents non‐asthma. Graphs show the estimated differences in outcomes variables and 95% confidence intervals (CI) when all exposures are at a particular quantile compared with when all are at the 25th or 50th quantile (D).

**FIGURE 3:**
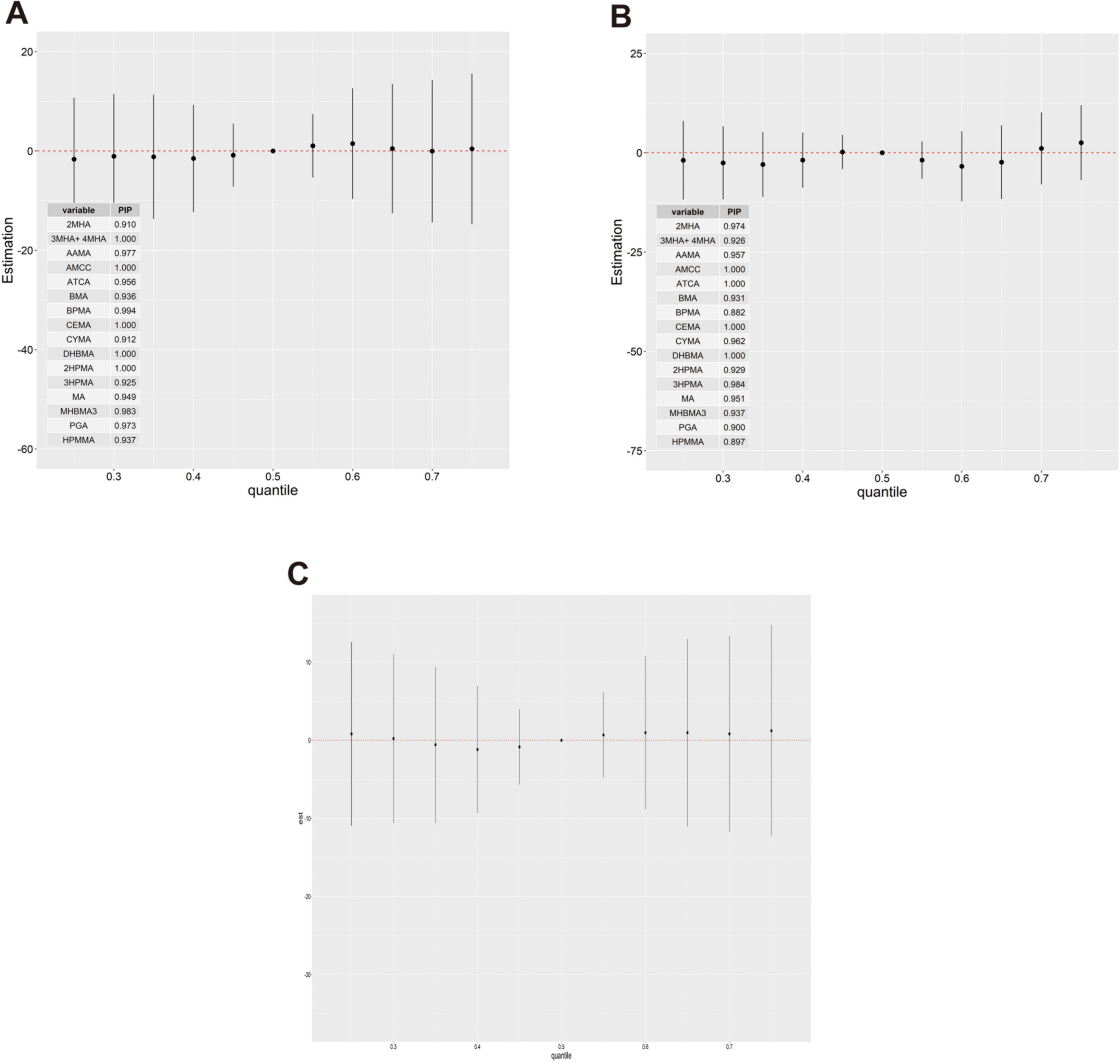
Associations of the UM‐VOCs with (A) asthma, (B) current asthma, and (C) wheeze by BKMR model adjusted for covariates as indicated in the text. Graphs show the estimated differences in outcomes variables and 95% confidence intervals (CI) when all exposures are at a particular quantile compared with when all are at the 50th quantile.

**FIGURE 4:**
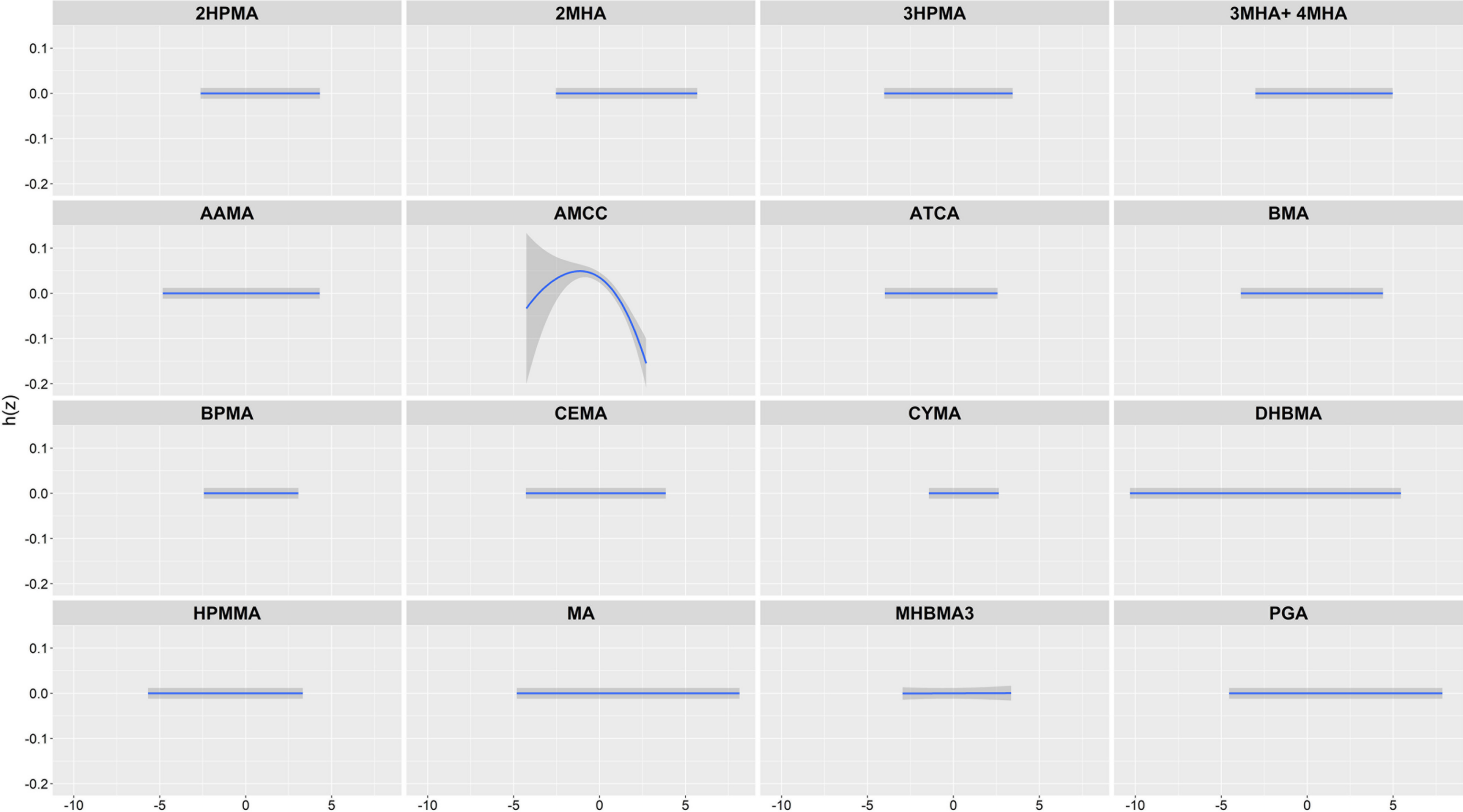
Univariate exposure–response curves and 95% CI interval for each UM‐VOC with the others fixed at the median.

Next, we estimated the overall effect of the UM‐VOCs mixture on FEV1, FVC, or FEV1/FVC (Figure 2A–C). As expected, we detected a negative relationship between the value of FEV1/FVC and the concentration of each of the UM‐VOCs mixture at or above the 60th percentile in comparison to the corresponding median urine level. And there was no overall difference in FEV1 and FVC between the median urinary concentrations of each of the 16 UM‐VOC mixtures and the urinary concentrations of each of the mixtures at certain percentiles (a range of 0.1 to 0.9 percentiles). And UM‐VOCs mixture was also revealed independent of asthma, current asthma, and wheeze across the entire range of exposure (Figure 3). In terms of FEV1/FVC, the AMCC was most important with a PIP of 1. Each PIP value for the remaining UM‐VOCs is approximately zero (Figure 2C). Corresponding with the abovementioned PIP values, for the relationship between individual UM‐VOC and FEV1/FVC, only AMCC showed a non‐monotonic association with FEV1/FVC, which was a decreasing trend in the higher concentration, while the remaining UM‐VOCs showed no relationships with FEV1/FVC when holding the other UM‐VOCs at their median values (Figure 4). Furthermore, our asthma‐specific analysis found evidence that exposure to VOCs is more strongly associated with FEV1/FVC among individuals with asthma compared with non‐asthma (Figure 2D).

**FIGURE 5:**
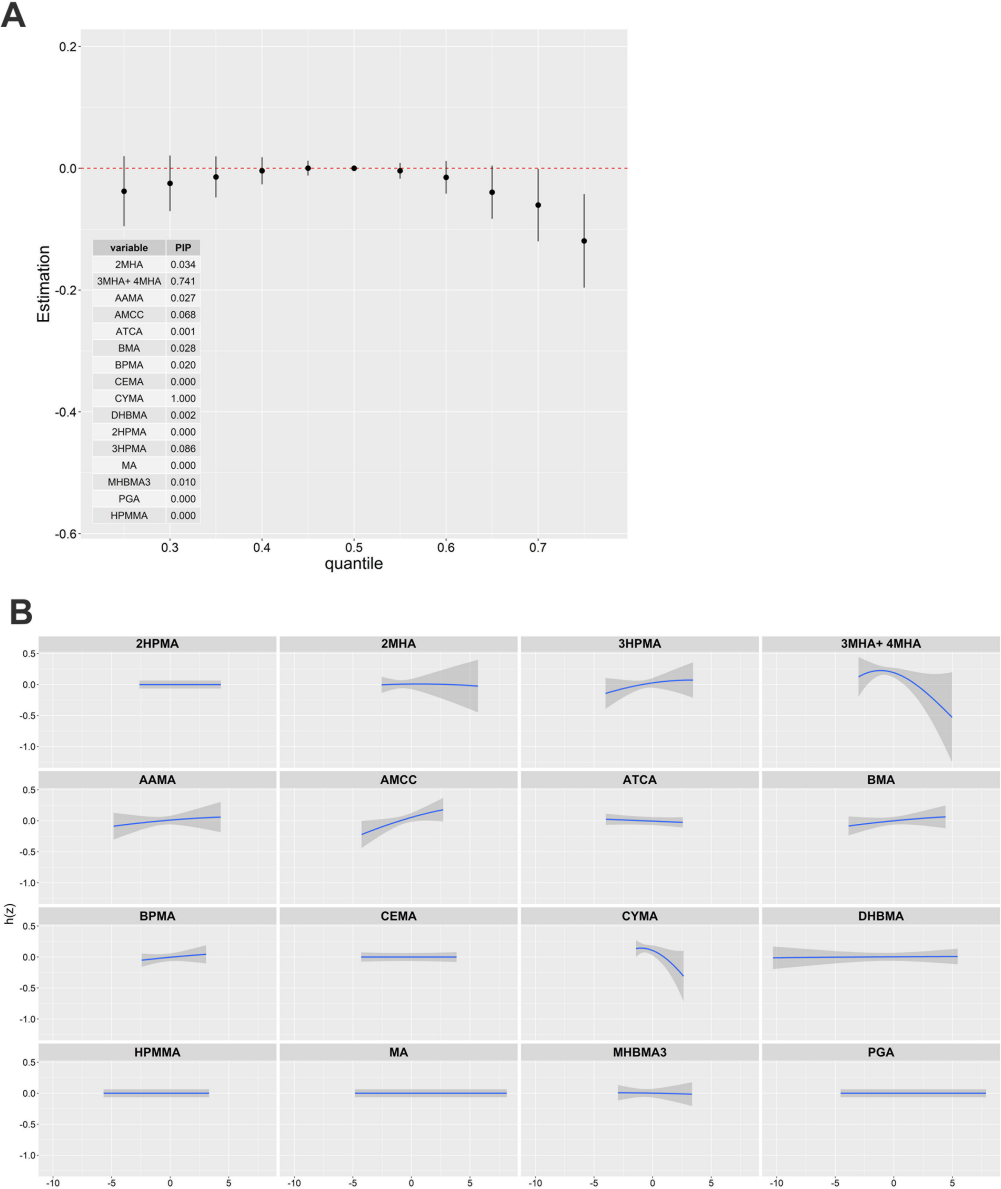
Overall effect of the 16‐UM‐VOC mixture on FeNO. (A) The UM‐VOCs mixture was associated with a significant decrease in FeNO when all chemicals were at or above the 70th percentile compared with their median values. (B) Univariate exposure–response functions show that CYMA and 3MHA + 4MHA demonstrated the strongest inverse associations with FeNO when other chemicals were fixed at their median values. Shaded areas represent 95% confidence intervals.

Next, we estimated the mixture's overall impact and exposure–response functions on FeNO (Figure 5). After controlling for the covariates, the concentrations of UM‐VOCs mixtures at or above the 70th percentile were negatively correlated with the values of FeNO compared with the corresponding 50th percentile. In the UM‐VOCs mixture, CYMA showed greatest importance with a PIP of 1 and then 3MHA + 4MHA with a PIP of 0.741, contributing most to joint effects, followed by 3HPMA and AMCC, with PIPs of 0.086 and 0.068, respectively, and PIPs of AAMA, BPMA, and BMA were all below 0.03. Figure 4B displays the exposure–response function trends for each of the 16 distinct chemicals. 3MHA + 4MHA and CYMA showed an obviously negative trend with FeNO in the highest concentration while 3HPMA, AAMA, AMCC, BPMA, and BMA showed a slightly positive trend with FeNO.

**FIGURE 6:**
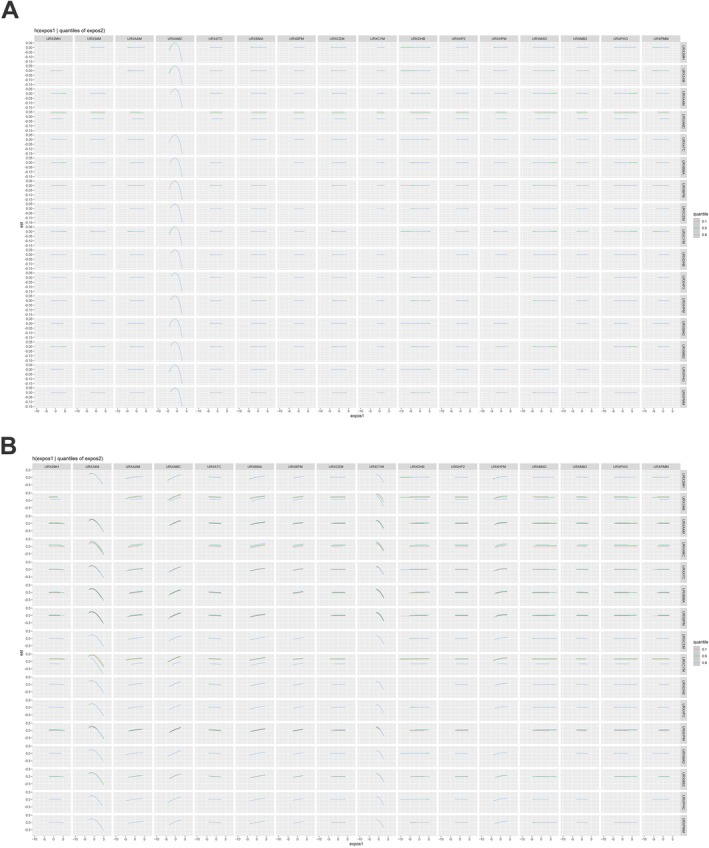
Bivariate exposure–response functions for 16 UM‐VOCs on (A) FEV1/FVC and (B) FENO when another chemical was fixed at varying (25th, 50th, and 75th) percentiles and all the other chemicals was fixed at the median.

Finally, we further examined the interactions between 16 UM‐VOCs (Figure 6). No evidence of interaction among these UM‐VOCs was shown as the slopes of the exposure–response lines remain relatively parallel.

### 3.4 The Potential Modifying Effects of Smoking on VOCs Exposure

**FIGURE 7:**
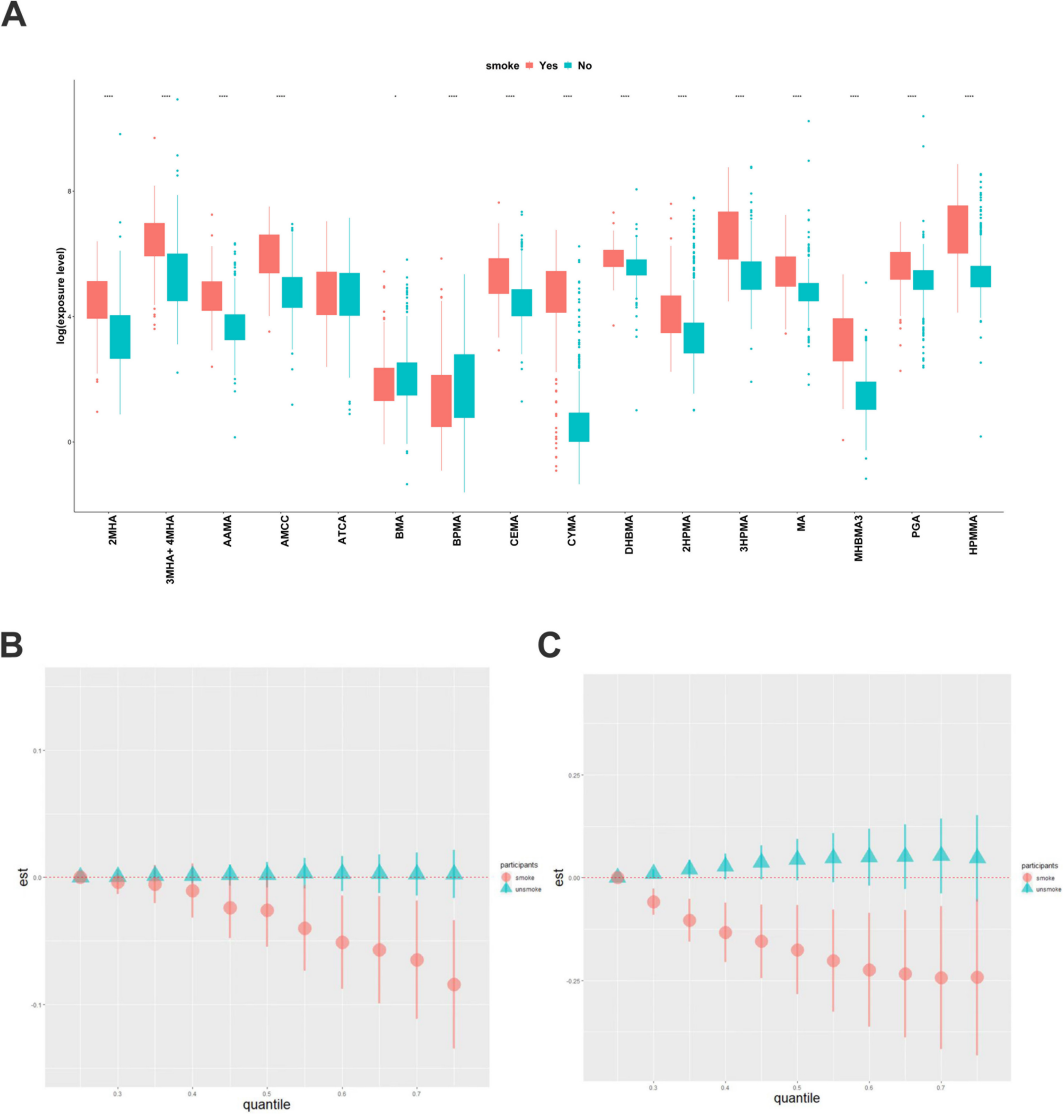
Differential exposure–response patterns by smoking status. (A) Concentrations of 13 out of 16 UM‐VOCs were significantly higher in smokers compared with nonsmokers (red = smokers, green = nonsmokers; Wilcoxon test *p* < 0.05). (B) The inverse association between the UM‐VOC mixture and FEV1/FVC was observed primarily in smokers, with little evidence of an association in nonsmokers. (C) For FeNO, distinct patterns were observed across subgroups: a negative association in smokers versus a positive association in nonsmokers when all chemicals were at higher percentiles compared with the 25th percentile. Shaded areas represent 95% confidence intervals.

To better understand the potential modifying effects of smoking, we first compared relative UM‐VOCs concentrations among smokers and nonsmokers because cigarette smoke is known to be an important source of VOCs. We found that, with the exception of ATCA, BMA, and BPMA, the levels of the remaining 13 compounds were significantly higher in smokers than in nonsmokers (Figure 7A, *p* < 0.05). Of note, the geometric means of CYMA were significantly higher in smokers, which are nearly zero among nonsmokers, followed by MHBMA3, HPMMA, AMCC, and AAMA.

Stratified BKMR analyses revealed differential associations by smoking status. The inverse association between the 16‐UM‐VOC mixture and FEV1/FVC was observed primarily among smokers, with little evidence of an association among nonsmokers (Figure 7B). However, because smokers may have lower baseline lung function compared with nonsmokers independent of VOC exposure, we cannot exclude the possibility that the stronger association in smokers reflects, at least in part, their poorer baseline respiratory health. Nevertheless, combined with the observation that most UM‐VOC concentrations were significantly higher in smokers (Figure 7A), our findings are consistent with the hypothesis that tobacco smoke may be a significant contributor to VOC exposure and that smokers may be more susceptible to VOC‐related lung function effects. Interestingly, when analyzing the effect of 16 UM‐VOCs on FeNO, we noticed that the trends went into opposite directions between smokers and nonsmokers (i.e., negative trend in smokers and positive trend in nonsmokers) (Figure 7C).

### 3.5 Data Analysis

**FIGURE 8:**
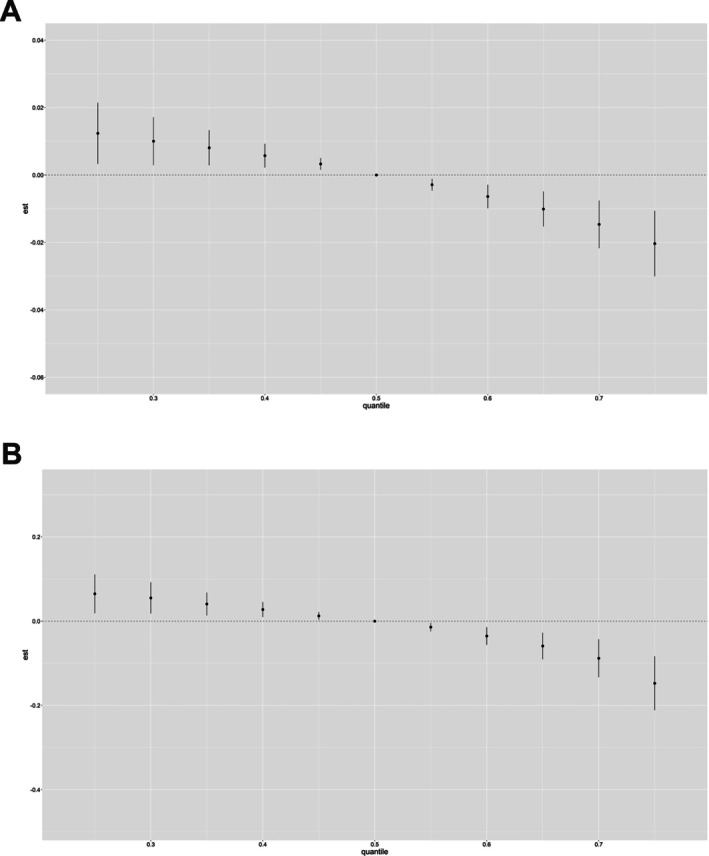
Associations of the UM‐VOCs with (A) FEV1/FVC and (B) FENO by BKMR before creatinine adjustment. Graphs show the estimated differences in outcomes variables and 95% CI when all exposures are at a particular quantile compared with when all are at the 50th quantile.

To evaluate the robustness of our conclusions, sensitivity analysis was performed by BKMR to assess the above correlations with or without normalization by urine creatinine. As shown in Figure 8, no much difference between the exposure–response curves before and after standardization.

## 4 Discussion

In the study, we found that exposure of the general population to environmental VOCs may impair lung function, and tobacco smoke may contribute to VOC exposure. To our knowledge, it is the first study that the BKMR model was used to explore the association between UM‐VOCs mixtures and lung function. Not only that, we further investigate FeNO, the marker of airway inflammation, and distinguish the contribution of each compound. As this study is cross‐sectional in design, we cannot directly investigate underlying biological mechanisms. The following discussion of potential mechanisms is primarily based on previously published literature and aims to provide context and plausible scientific explanations for our observational findings. These mechanistic interpretations should be considered speculative and require validation through future experimental studies.

Previous studies mainly focused on the detection of environmental samples, blood, or breath. Even though the accuracy of these samples was well‐validated by extensive observational and experimental studies, the high requirements for equipment or invasive operator‐dependent procedures limited their promotion and application [30]. To better circumvent these limitations, in this study, we sought to demonstrate the predictive potential of UM‐VOCs for lung function. The analysis of urinary metabolites might be easily operated, cost‐effective, and would be of wide public health significance. To the best of our knowledge, only a few studies have investigated the association between UM‐VOCs and lung function, with limits of focusing on only certain aspects [15, 31, 32, 33]. Our study provides a comprehensive understanding of the 16 common UM‐VOCs in terms of both joint and independent effects on multiple parameters of lung function, which is a valid complementation for previous findings.

In our analysis, linear regression identified inverse associations between several individual UM‐VOCs (AMCC, CYMA, MHBMA3, 3HPMA, AAMA, and MA) and FEV1/FVC (Table 2). When examined as mixtures using BKMR, the 16‐UM‐VOC mixture demonstrated a significant overall inverse association with FEV1/FVC at higher exposure percentiles (Figure 2C). Importantly, AMCC was identified as the dominant contributor within the BKMR framework (PIP = 1.0), consistent with its significant association in the single‐compound analysis. This convergence of findings from two complementary analytical approaches strengthens the evidence for a role of AMCC in lung function while the mixture analysis further revealed that the combined effect of multiple VOCs may exceed what would be expected from individual compounds alone. The convergence of findings across these two approaches strengthens the robustness of the observed associations. Although the specific molecular mechanisms explaining how VOCs interact with altered lung function remain largely unclear, previous studies have proposed several plausible pathways. For example, experimental evidence suggests that VOC exposure may induce glutathione depletion and aberrant reactive oxygen species (ROS) production, leading to oxidative stress, lipid peroxidation, and oxidative DNA damage [34]. Besides this, it is suggested that VOCs exposure may alter air–liquid interface properties of surfactants in the lungs [5]. However, whether these mechanisms underlie the associations observed in our study cannot be determined from our data and requires further investigation.

However, such a trend was not observed for FEV1 and FVC in our current study. This finding fits into a previous study where Yoon et al. discovered that FVC did not show any association with markers of VOCs exposure compared with FEV1/FVC [33]. In particular, our BKMR analysis identified urinary AMCC as the dominant contributor to the mixture effect on FEV1/FVC (PIP = 1.0, Figure 2C). Similar to our findings, a cohort study found that urinary AMCC is dose‐dependently associated with longitudinal lung function decline [31]. Additionally, the BKMR model confirmed that smokers and asthma individuals are more susceptible to VOCs‐induced lung injury. Several studies have linked VOC exposure to asthma and other respiratory symptoms [35]. Recent evidence from a systematic review and meta‐analysis further supports that VOC exposure is associated with impaired pulmonary function and increased respiratory symptoms [36]. According to Zock et al., the frequent exposure to VOCs might result in a 40% rise in wheeze and a 50% increase in asthma symptoms [37]. A similar study from NHANES 2005–2006 also found that higher acrylamide, the parent compound of AAMA, exposure was significantly associated with an increased risk of asthma by 53% in females and wheeze by 65% in adults [38]. Our study also showed individually positive relevance of 3HPMA and HPMMA to wheeze. However, in the current study, no statistically significant change in asthma or current asthma was observed either in linear regression or BKMR model analysis. This may be partially attributable to the small sample size of symptomatic individuals, which might have led to insufficient statistical validity.

FeNO associated with eosinophilic inflammation was used to measure the severity of T‐helper cell type 2 (Th2) allergic inflammation in the airways, reflecting underlying airway inflammatory processes [39]. BKMR analysis indicated the negative association of UM‐VOCs at high concentrations with FeNO and the opposite but non‐significant trend at lower concentrations compared with the 50th percentile. Urinary CYMA, a metabolite of acrylonitrile, was identified as the most influential contributor to the decrease in FeNO from our analysis. But there are no relevant studies on the mechanism of VOCs contributing to the FeNO change. Inflammatory theory seemed not enough to explain our study, as it needs to be noted that the relationship between exposure to pollutants and adverse respiratory effects may involve both allergic and nonallergic pathways [40]. For UM‐VOCs, both mechanisms may be in play [35, 41]. The FeNO may not properly reflect Th1 inflammation, and low levels of FeNO can be accompanied by elevated neutrophil counts [42]. Additionally, COPD is not discriminated well in our study, in which FeNO has been reported to be highly variable [43]. Worth mentioning, our research assessed the modification effect of smoking on the association of UM‐VOCs with lung function parameters by BKMR. Before this, we compared UM‐VOCs concentration in smokers and nonsmokers. It was no surprise that smokers had higher levels of most VOC metabolites than nonsmokers, which was in accordance with previous papers [1, 21, 44]. As the UM‐VOCs concentration increased, FEV1/FVC decline appears only in smokers. Regarding this, we offered two possible hypotheses: First, smoking‐increased susceptibility to lung injury was caused by VOCs, and second, tobacco smoke was the main source of VOCs. Integrating the results from our study, the latter may play the more important role; however, our conclusion remains to be further verified by other population‐based or experimental studies. Interestingly, when analyzing the effect of 16 UM‐VOCs on FeNO, we noticed that the trends went in opposite directions between smokers and nonsmokers.

According to Kharitonov et al., smokers' FeNO levels were more than 50% lower than nonsmokers' levels [45]. This may be a result caused both by the decrease of NO formation, evidenced by reduced inducible nitric oxide synthase (iNOS) activity in lung epithelial cells [46], and conversion of NO into peroxynitrite induced by increased ROS stimulated by smoking [45]. We therefore speculated reasonably that the effects of VOCs and cigarette smoke overlapped to a large degree. As for nonsmokers, the opposite trends could arise from special UM‐VOCs (i.e., ATCA, BMA, and BPMA), which were higher to some degree in the nonsmokers. Because the VOCs exposure profiles differed obviously in smokers and nonsmokers, and some VOCs could discriminate between Th1‐ and Th2‐type inflammation [47], while FeNO was associated with Th2 inflammation only, indicating some unclear mechanisms involved in smoke‐related lung damage, further research is required to test our conjecture.

We acknowledge some limitations of this study. First, the cross‐sectional design could not effectively conclude reasonable causal interpretations. Second, we used self‐reported asthma rather than a more accurate clinical diagnosis for disease classification. Third, we cannot be certain that UM‐VOCs reflect true VOCs exposure in the ordinary environmental circumstances. Fourth, we did not examine how other significant pollutants interacted with alterations in lung function, such as polycyclic aromatic hydrocarbons, for the lack of such information. Fifth, we evaluated only an annual cycle and the sample size was insufficient due to the higher missing rates of covariates. Finally, as the study population was restricted to adults aged 20 years or older, our results cannot be extrapolated to entire populations.

## 5 Conclusions

In a population‐based survey, we established a link between urinary metabolites of volatile organic compounds (UM‐VOCs) and lung health in adults. Using BKMR, we observed a joint effect of 16 UM‐VOCs on decreased lung function. Our findings suggest that exposure to ambient VOCs in the general population may be associated with reduced lung function, with tobacco smoke presenting as a possible contributor to VOC exposure. Larger longitudinal studies and laboratory investigations are warranted to validate our findings.

## Author Contributions

Qian Liu and Yanpeng Fu wrote the article; Wenyu Chen contributed to the data analysis; Yuchen Tao and Yue Wang reviewed and edited the article; Haiyan Yin and Qianqian Yang contributed to designing the study.

## Funding

This work was supported by the Research Program of Jiangsu Provincial Health Commission (MQ2025022).

## Ethics Statement

The authors have nothing to report.

## Conflicts of Interest

The authors declare no conflicts of interest.

## Supporting information


**Figure S1:** Flow diagram of participants for the study (NHANES 2011–2012).
**Table S1:** Detectable frequencies of 16 urinary VOCs metabolites and their parent compounds.
**Table S2:** Association of the concentrations of urinary VOCs metabolites with asthma and current asthma (linear regression models).

## Data Availability

The data that support the findings of this study are available in NHANES (https://www.cdc.gov/nchs/nhanes/index.htm). These data were derived from the following resources available in the public domain: NHANES 2011–2012 dataset (https://www.cdc.gov/nchs/nhanes/).
