# Supplementary material for: Association Between Volatile Organic Compound Metabolites in Urine and Adult Lung Function: Evidence From NHANES 2011–2012
Source: Clin Respir J. 2026 May 1;20(5):e70192. doi: 10.1111/crj.70192 (PMC13133596; doi:10.1111/crj.70192)
Supplement: Supplementary file 1 — Figure S1: Flow diagram of participants for the study (NHANES 2011–2012). Table S1: Detectable frequencies of 16 urinary VOCs metabolites and their parent compounds. Table S2: Association of the concentrations of urinary VOCs metabolites with asthma and current asthma (linear regression models). [file CRJ-20-e70192-s001.docx]

**Figure Legends**

**Figure S1.** Flow diagram of participants for the study (NHANES 2011–2012).

**

**

**Table S1.** Detectable frequencies of 16 urinary VOCs metabolites and their parent compounds

| Parent compounds | Urine metabolites | Abbreviated names | Detectable frequencies |
| --- | --- | --- | --- |
| Xylene | 2-Methylhippuric acid | 2MHA | 92.59% |
| Xylene | 3- and 4-Methylhippuric acid | 3MHA+ 4MHA | 99.60% |
| Acrylamide | N-Acetyl-S-(2-carbamoylethyl)-L-cysteine | AAMA | 99.90% |
| N,N-Dimethylformamide | N-Acetyl-S-(N-methylcarbamoyl)-L-cysteine | AMCC | 99.65% |
| Cyanide | 2-Aminothiazoline-4-carboxylic acid | ATCA | 96.56% |
| Toluene | N-Acetyl-S-(benzyl)-L-cysteine | BMA | 99.35% |
| 1-Bromopropane | N-Acetyl-S-(n-propyl)-L-cysteine | BPMA | 75.20% |
| Acrolein | N-Acetyl-S-(2-carboxyethyl)-L-cysteine | CEMA | 99.25% |
| Acrylonitrile | N-Acetyl-S-(2-cyanoethyl)-L-cysteine | CYMA | 86.85% |
| 1,3-Butadiene | N-Acetyl-S-(3,4-dihydroxybutyl)-L-cysteine | DHBMA | 99.98% |
| Propylene oxide | N-Acetyl-S-(2-hydroxypropyl)-L-cysteine | 2HPMA | 94.87% |
| Acrolein | N-Acetyl-S-(3-hydroxypropyl)-L-cysteine | 3HPMA | 99.84% |
| Styrene | Mandelic acid | MA | 98.59% |
| 1,3-Butadiene | N-Acetyl-S-(4-hydroxy-2-butenyl)-L-cysteine | MHBMA3 | 96.75% |
| Ethylbenzene, styrene | Phenylglyoxylic acid | PGA | 99.23% |
| Crotonaldehyde | N-Acetyl-S-(3-hydroxypropyl-1-methyl)-L-cysteine | HPMMA | 99.99% |

**Table S2.** Association of the concentrations of urinary VOCs metabolites with asthma and current asthma. (linear regression models)

| Metabolites | Asthma | | Current asthma | |
| --- | --- | --- | --- | --- |
|  | β | Adjusted P value | β | Adjusted P value |
| 2MHA | -0.1751391 | 0.34026593 | -0.2466165 | 0.1819749 |
| 3MHA+ 4MHA | -0.08614115 | 0.62230922 | -0.1792461 | 0.3474086 |
| AAMA | 0.1911705 | 0.36506234 | 0.0224188 | 0.9199260 |
| AMCC | 0.2732151 | 0.22281440 | 0.08655685 | 0.7814419 |
| ATCA | 0.04396149 | 0.79059067 | -0.001432533 | 0.9932803 |
| BMA | 0.1134104 | 0.28220599 | 0.1544352 | 0.3770839 |
| BPMA | 0.134882 | 0.13262794 | 0.1627319 | 0.2149155 |
| CEMA | 0.1936576 | 0.41036692 | 0.2247326 | 0.4429020 |
| CYMA | 0.05603409 | 0.64790179 | -0.01249306 | 0.8988342 |
| DHBMA | 0.4359554 | 0.30464235 | 0.5695049 | 0.2190591 |
| 2HPMA | -0.02738608 | 0.82306367 | -0.1377756 | 0.4231884 |
| 3HPMA | 0.3391285 | 0.07589192 | 0.2986269 | 0.2410277 |
| MA | 0.3873592 | 0.15540805 | 0.4168854 | 0.2104584 |
| MHBMA3 | 0.04407953 | 0.80638329 | -0.06394008 | 0.7067732 |
| PGA | 0.08521748 | 0.75049955 | 0.2037152 | 0.4840886 |
| HPMMA | 0.2507141 | 0.14120513 | 0.2468558 | 0.2603226 |
